# The influence of stigma on PrEP uptake among adolescent girls and young women in Johannesburg, South Africa and Mwanza, Tanzania: Qualitative findings from the EMPOWER study

**DOI:** 10.1371/journal.pone.0339380

**Published:** 2026-02-03

**Authors:** Anne L. Stangl, Caitlin Bryan, Iman Barré, Lethabo Ramskin, Shelley Lees, Nomhle Ndimande-Khoza, Deborah Baron, Manuela Colombini, Sheila Harvey, Saidi Kapiga, Fiona Scorgie, Sinéad Delany-Moretlwe

**Affiliations:** 1 International Center for Research on Women, Washington, District of Columbia, United States of America; 2 Department of International Health, Johns Hopkins University, Baltimore, Maryland, United States of America; 3 Wits RHI, Faculty of Health Sciences, University of the Witwatersrand, Johannesburg, South Africa; 4 London School of Hygiene and Tropical Medicine, London, United Kingdom; 5 Mwanza Intervention Trials Unit, Mwanza, Tanzania; Beth Israel Deaconess Medical Center/Harvard Medical School, UNITED STATES OF AMERICA

## Abstract

**Background:**

Adolescent girls and young women (AGYW) in sub-Saharan Africa need HIV prevention options. Stigma may impede uptake and consistent use of oral pre-exposure prophylaxis (PrEP). We sought to understand how stigma manifested and influenced decisions of AGYW participating in a PrEP demonstration trial in Johannesburg, South Africa and Mwanza, Tanzania.

**Methods:**

This paper reports on serial qualitative in-depth interviews (IDI) with AGYW (n = 39) as well as healthcare providers and community stakeholders (n = 30) conducted during a randomized controlled trial that evaluated the effect of empowerment clubs to address harmful gender norms and stigma on PrEP persistence in AGYW aged 16–24 in South Africa and Tanzania. Analysis was thematic and inductive.

**Results:**

Stigma manifested in two main ways: HIV-related stigma and sexual behavior stigma. PrEP was often mistaken for antiretroviral therapy, and some participants taking daily PrEP reported that partners or family members thought they were living with HIV. Anticipated PrEP sexual stigma was common and linked with concerns around AGYW being perceived as promiscuous. While most participants anticipated stigma related to PrEP use, experiences of stigma were rare and did not deter AGYW from initiating PrEP. Many participants demonstrated resilience and remained confident in their decision to use PrEP and some developed strategies to avoid stigma, such as hiding pills or taking PrEP when partners or family members were not around.

**Conclusions:**

Our findings suggest that anticipated stigma is a common yet surmountable concern of AGYW initiating PrEP in eastern and southern Africa. Future PrEP implementation should be paired with multi-level activities to reduce stigma including empowerment activities for adolescents, strategies to reduce negative attitudes among health care providers and community-wide education to raise awareness and position PrEP as a responsible choice for young people to protect their health.

**Trial registration:**

Pan African Clinical Trials Registry PACTR202006754762723.

## Introduction

Adolescent girls and young women (AGYW) in eastern and southern Africa (ESA) are increasingly vulnerable to HIV [1]. Structural factors, such as gender inequality, poverty, exposure to violence and low social power contribute to the high risk of HIV experienced by AGYW [2]. In South Africa, HIV prevalence among AGYW aged 15–24 years old is four times higher than male peers [3]. Similarly, in Tanzania, young women have a three-fold higher prevalence of HIV than their male counterparts [4]. Despite their increased vulnerability to HIV, AGYW in ESA have had few prevention options fully within their control prior to the advent of oral pre-exposure prophylaxis (PrEP). In 2015, the World Health Organization (WHO) recommended oral pre-exposure prophylaxis as part of a comprehensive HIV prevention package for individuals at substantial risk for HIV acquisition [5]. Countries including South Africa and Tanzania, supported demonstration projects of oral PrEP delivery prior to specific populations prior to national expansion from 2019 onwards [6]. Despite expanding access to oral PrEP, persistence remains a challenge with 43% of women discontinuing within six months of initiation [7].

Stigma refers to a negative social label applied to a person or group that results in shame, prejudice or discrimination [8]. At its core, HIV stigma stems from moral judgements made about behaviors deemed socially unacceptable, such as promiscuity or injection drug use, that are perceived to lead to HIV acquisition [9]. Initial oral PrEP trials in AGYW in ESA showed that fear of experiencing HIV-related stigma undermined daily pill-taking, with antiretroviral (ART) pills being associated with illness [10,11]. PrEP use may also be viewed as encouraging perceived risky sexual practices, like condomless sex or having multiple partners, exposing users to moral scrutiny and stigma, particularly if they are young women [9]. Stigma is a well-documented barrier to health-seeking, engagement in care, and adherence to treatment across a range of health conditions, including HIV [12]. When linked to PrEP use, stigma may significantly limit uptake and persistence undermining the potential benefits of oral PrEP. Formative research carried out in Nigeria identified stigma as a major concern and barrier to PrEP uptake and use [13]. Similarly, a study carried out in the U.S. among women found that intent to use PrEP was lower among those who anticipated stigma and disapproval [14]. Findings from a qualitative study conducted among healthcare providers (HCP) in local clinics in South Africa also showed that stigma within health clinics presented a significant barrier for AGYW taking oral PrEP [15].

The EMPOWER study evaluated the impact of an empowerment intervention that addressed stigma and harmful gender norms among AGYW aged 16–24 years in South Africa and Tanzania on PrEP persistence (PACTR202006754762723). The purpose of this analysis is to understand how PrEP stigma was defined and experienced among AGYW enrolled in the EMPOWER study. We focused on the diverse consequences of anticipated and experienced stigma, as well as the role of community beliefs, interpersonal relationships within families and intimate partners, and healthcare provider interactions.

## Materials and methods

### Study design and population

This qualitative study was part of EMPOWER, a trial that evaluated the effectiveness of a four-session empowerment curriculum aimed at addressing stigma and harmful gender norms delivered as part of monthly adherence support clubs compared to standard of care adherence support on PrEP persistence. A total of 431 sexually active, non-pregnant AGYW aged 16–24 years at the time of enrolment, without HIV and interested in initiating oral PrEP in Johannesburg, South Africa and Mwanza, Tanzania were enrolled between August 2016 and February 2018, and followed up for 6–15 months. PrEP initiation was not a pre-requisite for enrolment, and PrEP acceptance and persistence were the primary outcomes of interest. Participants attended quarterly clinic visits for HIV testing and PrEP refills if required. A subset of participants was invited to participate in up to three serial in-depth interviews (IDI). The target sample size for IDI was 25 per site. In addition, interviews with HCP and community stakeholders in each site were planned to assess attitudes to PrEP. At the time, oral PrEP access outside of study settings was limited and largely unheard of in both countries. The study was reviewed and approved by the Human Research Ethics Committee of University of the Witwatersrand (M151199) and the Mwanza Intervention Trials Unit. All participants provided written informed consent prior to study enrollment.

### Study setting

Participants in Johannesburg, South Africa were recruited from the inner-city neighborhood of Hillbrow and its surrounds. The area is characterized by dense, over-crowded high-rise residential buildings and abandoned industrial buildings informally occupied by migrants from South Africa and the region. Several schools and tertiary education institutions are located in the district, and many inhabitants reside in the area because it is close to school or work. The study clinic is located adjacent to a youth-friendly clinic that provides services for 10–24-year-olds.

Mwanza is Tanzania’s second largest city, located on the shores of Lake Victoria. Opportunities for poorly educated women are often limited to informal work in small businesses or more formal work in bars and guesthouses, from which many study participants were recruited. While female bar workers who serve food and alcohol at bars may not self-identify as sex workers, they face a heightened risk of HIV infection due to factors like transactional sex to supplement income, frequent or recurrent sexually transmitted infections, and limited ability to negotiate condom use [16].

### Qualitative data collection

Initially participants were randomly selected to participate in the qualitative subset. Midway through recruitment, the sampling strategy was adjusted to capture a broader range of experiences and to ensure the qualitative cohort reflected the larger study sample, which at that point included participants who: 1) had declined PrEP at enrollment, even if they subsequently accepted; 2) had disclosed gender-based violence at screening; 3) were in the age group 18–20. The first round of interviews took place within three months of PrEP initiation. This interview focused on assessing motivations for uptake, and initial experiences and challenges with taking PrEP. Second round interviews were held at around six months after enrollment and focused on late barriers to adherence. The final round was conducted between nine and twelve months after PrEP initiation and assessed experiences of adherence and participants’ perspectives on the study interventions. In Tanzania follow up was restricted to six months for operational reasons and only two interviews were completed. Key informant interviews (KII) were conducted with purposively selected community stakeholders and HCP from the EMPOWER study at study end.

Detailed interview guides were developed by the research team. All IDIs were conducted by trained interviewers in the preferred language of the participant (English, isiZulu, seSotho and Swahili). All KII were carried out in English. Interviews were conducted in a private room and took about 45–60 minutes. All IDIs, except one, were audio-recorded, transcribed verbatim, and translated into English where necessary. In one IDI, a participant did not give consent for the audio-recording; hand-written notes were taken instead and later transcribed. Quality and accuracy of transcriptions were checked by interviewers against the recordings. Lastly, observation notes by study staff from three EMPOWER club sessions on stigma and discrimination in South Africa were used to inform our analysis.

### Data analysis

Analysis followed a thematic, inductive approach with key themes identified directly from the interview data. Interpretation of the emerging data was discussed and specific themes identified in two analysis workshops involving the qualitative study team. A provisional codebook was developed following the open coding of a small selection of transcripts by qualitative team members. Transcripts were coded in NVivo 11. Consistency of coding decisions was checked using a process in which two members of the team independently coded the same transcript and inter-coder reliability (ICR), set at.75, was assessed. Thereafter the codebook was finalized, and the remainder of the interview data was inductively coded by four coders. Reports were generated for specific nodes and summary matrices developed to examine intersecting themes.

Coded transcripts were then analyzed by US-based team members (AS, CB, IB) for stigma-related themes because of their expertise and interest in stigma specifically [8]. The codes analyzed in Round 1 IDIs were: community talk about PrEP, stigma, influences taking PrEP, reasons for taking or declining PrEP, clinic experiences, youth and AIDS, personal experience, HIV testing and disclosure. Rounds 2 and 3 IDIs were combined and analyzed for stigma themes in the following codes: community talk about PrEP, stigma, influences taking PrEP, clinic experiences, disclosure, adherence, and discontinuation. Additionally, the healthcare provider and community stakeholder KIIs were analyzed for stigma themes. Full transcripts were reviewed for further clarity if needed. For Tanzania, a complementary codebook was developed to capture stigma-related themes by the US-based team and used to code the IDI and KII transcripts. The sub-codes included: HIV stigma, sexual behavior stigma, sex work stigma, anticipated PrEP stigma, and experienced PrEP stigma. In the analysis, we explored the relationship between anticipated stigma and experienced stigma; and whether participants who had anticipated or feared stigma from their use of PrEP had actually experienced stigma as a result of their PrEP use. This led to explorations of potential impacts on relationships, as well as consequences of PrEP use. Additionally, we explored themes of resilience and how participants may have navigated their use of PrEP despite their fears or experiences of stigma.

## Results

### Participant characteristics

A total of 39 AGYW participants (South Africa n = 25, Tanzania n = 14) were interviewed on one or more occasions. In South Africa nine women completed three interviews, 12 completed two interviews and four completed one interview (n = 55 interviews), while in Tanzania 12 participants were interviewed twice and two were interviewed once (n = 26 interviews). Additionally, there were 16 KII with HCP and 14 KII with community stakeholders.

Despite being of similar age, the adolescent study populations in South Africa and Tanzania were quite different, not only in their occupations and circumstances of work, but also in their uptake and use of PrEP. In South Africa, most participants were tertiary-level students living with family or in student residences. Eighteen reported being in a relationship, three described themselves as “dating”, three identified as single, and one did not specify her relationship status. In Tanzania, 14 participants between the ages of 20 and 25 years were interviewed. Thirteen of the participants worked at bars, hotels, or cafes and occasionally engaged in paid sexual transactions with customers. The presentation of our findings takes these differences into account, and findings are described separately by country.

### Anticipated stigma

Participants in both locations anticipated stigma related both to HIV and to sexual activity. When asked about anticipated stigma related to their PrEP use, participants in South Africa located two main sources from which they anticipated stigma to come: from their communities and from their parents. Communities were often described as including church members, schoolmates or peers, as well as neighbors. Participants were less worried about experiencing stigma from their peers, whom they felt were more supportive, understanding of their life circumstances, and excited to learn more about or even take PrEP themselves. Many participants expressed concern about what adult community members might say if they were identified as using PrEP. They feared being perceived as having HIV or being labeled as promiscuous for using PrEP. Participants anticipated that PrEP would be mistaken for HIV medication and, therefore, feared they would experience HIV-related stigma. There was a general sense that communities, family members, and partners lacked knowledge about PrEP, which could lead to false assumptions that PrEP was in fact HIV treatment.

*“They would say I’m HIV positive, because some people don’t understand anything about PrEP.” (18-year-old, baseline, South Africa)*

Study participants also anticipated that taking PrEP would be associated with perceptions of promiscuity.

*“Why is she using PrEP, it means she is sleeping around. Why? You know, such comments.” (18-year-old, baseline, South Africa)*

Participants expected mixed reactions from parents and adult relatives about taking PrEP. Some participants described supportive parents that encouraged active steps toward health promotion and HIV prevention and would approve of them taking PrEP, whereas others feared that parents would think that they were behaving inappropriately, as AGYW should not be having sex at all. Many participants described a fear of disclosing PrEP use to parents due to anticipated stigma associated with perceptions of promiscuity. Anticipated HIV-related and sexual behavior-related stigma often overlapped and influenced the willingness of AGYW to disclose PrEP use. One participant did not want to disclose to her mother that she was taking PrEP because her mother believed she was a virgin. Additionally, this participant believed that the community would think she was HIV-positive if they knew she was taking PrEP.

*“It creeps me out, it makes me scared, I mean my parents are well-known people. They are respected in the society, so when that kind of rumor spreads, I mean I would die, before they would kill me, I would die first.” (18-year-old, baseline, South Africa)*

In Tanzania, stigma related to sex work, and to sexual behavior, was also mentioned across all interviewed groups. Participants and key informants suggested that some of the participants were involved in sex work or transactional sex at the bars or hotels where they worked.

*“I worried that if my parents or family members find out that I am using those medicines [PrEP], maybe they would think of me as... a prostitute as the reason for my use of them.”* (*25-year-old, baseline, Tanzania*)

*“In the society she may be perceived as someone who partakes in much sexual intercourse. Yes, therefore these things do exist and for those who hide, they tell you openly that they don’t want anyone to know about it.”* (*HCP, Tanzania)*

The nature of these activities was complex, as some of the participants described coercive work environments that necessitated sexual engagement with clients as a condition of payment or employment.

*“Yes, they are affected because one may stigmatize herself; she may see that she already has been considered a whore since she stays at the bar; these people already think she is immoral. So, they are affected psychologically. She begins to stigmatize herself; even if I have multiple men, I don’t care, because they already said I’m a whore and I work at a bar.”* (*HCP, Tanzania*)

Concerns around HIV stigma because of the similarity to the ARV pill bottles were also discussed among participants in Tanzania.

*“Maybe when they see the tins resemble with tins for HIV/AIDS tablets they think they are tablets for HIV/AIDS...If you see how the tin is...It looks similar. But the tablets are not similar.” (24-year-old, endline, Tanzania)*

### Experienced stigma

The data revealed diverse consequences of experienced stigma, including fear of disclosure, shame, embarrassment, judgement, name-calling and gossip, leading to missed doses. Partners and parents often actively discourage PrEP use, although instances of resilience were observed. One consequence of social norms that define sexual activity among AGYW as ‘bad’ or ‘inappropriate’ is that access to sexual and reproductive health services becomes restricted.

*“…Let’s say you want to do an HIV test, they [healthcare workers] will ask,* ***at this age, ‘why do you want to do an HIV test? You are still young.****’ Which is not nice, and which is not fair, it makes you feel embarrassed and then you just decide to go [so] you do not know your health status and where you are standing, uhum.” (22-year-old, baseline, South Africa)*

Community stakeholders endorsed the observation that some health care workers were judgmental of sexually active adolescents.

*“ …back then when I started working in 2015, I saw how [AGYW] were treated, more especially these ones for family planning. They used to be discriminated upon. They used to, some of them, nurses felt that they are too young to come forward about them. So, with PrEP, I have never heard stories of them being judged, but I think it might be the situation because sometimes healthcare providers think that adolescents don’t have even [rights], though we know they do have rights, but sometimes we tend to forget. [Healthcare providers] tend to forget and judge.” (Community stakeholder, South Africa)*

Some South African participants described that experienced stigma led to their reasons for taking PrEP and participating in the study being dismissed or disbelieved, particularly by men.

*“With me with the people that I have told so far, they think that I am joking, especially guys, they will be like ‘you are joking’. With others, when I tell them, what comes to their mind is it means that I will be promiscuous, you will go sleeping around. People who understand me are my peers, my generation, they understand me, it’s PrEP, PrEP, because most of my friends, we talk the same language, we take PrEP.” (19-year-old, baseline, South Africa)*

Others described the impact PrEP use was having on their relationships with intimate partners. They explained that their partner’s perceptions of PrEP, or even taking medicine generally, introduced strain into the relationship, and feelings of insecurity.

*“We agreed but in deep I knew, he doesn’t want a person who was going to drink a pill every day, I want someone who is normal.” (21-year-old, baseline, South Africa)*

One participant’s partner believed that she was HIV positive because the pamphlet for PrEP had the words “HIV-1” written on them. He would hide the pills from her, and this would sometimes result in her missing a dose.

*“Okay, by those times neh, he would like take my pills and hide them for me.” (21-year-old, endline, South Africa)*

A HCP mentioned that parents had also been known to discard PrEP pills.

*“Some of them, it’s not their partners who throw away pills…it’s their parents, who would say, ‘are you sexually active because you are saying that this is for the high risk person, so you are telling me that you are a high risk, you have more than one partner’, you know.” (Healthcare provider, SA)*

During an Empower Club session on stigma, one participant shared her experience of discontinuing use because of her mother not being happy with her taking PrEP.

*“Another participant said though she was still in the study, she had stopped taking PrEP because her mother was not happy with her taking PrEP.” (EMPOWER Club session observation notes)*

In Tanzania, participants described HIV-related stigma based on the resemblance of the PrEP container to the containers used for ARVs.

*“When I could go and pick up the containers or even when I started, [family members might say]: ‘she has brought her containers’ (In Sukuma). It’s that she has also carried her containers; I don’t know where she is going with her HIV.” (20-year-old, endline, Tanzania)*

Violence from intimate partners triggered by the assumption that PrEP use signified living with HIV was reported exclusively in Tanzania.

*“He may even beat me up. It’s because those who do not know those medicines [PrEP], think simply that they are for HIV infected patients.” (20-year-old, endline, Tanzania)*

*“She told me, ‘My boyfriend forcibly took away the medicine container from me.’*

*Eeh, therefore when he sees her using it, he directly thinks that she is infected.”* (*HCP, Tanzania*)

### Influence of stigma on PrEP initiation and continuation

Qualitative analysis revealed resilience of two kinds: 1) strategies used to avoid stigma so that PrEP use could be more feasible, as well as 2) the use of PrEP despite stigma. One HCP in South Africa described the lengths some participants would go to hide their PrEP use to avoid being the subject of gossip and judgment. This included hiding the pills, as well as concealing pill taking by making sure they were alone.

*“Ja, when you come to that because some of them would have…they have a problem with going with the container because it’s written Truvada. Stigma is an issue for them, you know. Somebody who is not having a broader mind, who doesn’t understand what PrEP is, they have to explain to everyone that no, this is not an antiretroviral, something that is going to prevent me from getting HIV and then they have to hide the pill. They need to decant the pills, they need to put them in tissues because they are afraid that people might judge them, you know. They will say that the pills got lost because they were wrapping them with the tissue***,** *they are decanting them from the container to the tissue.” (HCP, SA)*

One participant adopted strategy to mitigate stigma by postponing PrEP use until the relatives she lived with were no longer present, thereby avoiding exposure to their judgmental attitudes.

*“[One participant], she declined PrEP at the beginning because she was staying with her two aunts and according to her, her two aunts are judgmental, so she started taking PrEP later because her aunts were now in [KwaZulu-Natal] KZN, she is no longer staying with them. That is when she felt now is a good time for me to take PrEP.” (HCP, SA)*

Participants in Tanzania were adamant regarding their commitment to using PrEP and were not dissuaded by societal pressures to stop taking PrEP. Participants cited various reasons for not telling others they were using PrEP, including poor timing, fear of backlash, or not feeling it was necessary, among others. However, all participants were confident in their decision to use PrEP and felt empowered by the education they had received about its health benefits. Many participants cited their risky, and often dangerous, work environments as their impetus to protect their health by using PrEP. Many believed that their peers and family members would accept their decision to use PrEP once they were also educated about it. Some even stated that their peers would be interested in using PrEP should it become available, suggesting that anticipation of stigma did not prevent them from initiating PrEP.

*“R: No, I didn’t have fear, I just found myself confident to even elaborate to a person, and I don’t let myself to have fear anymore.*

*I: Why didn’t you think of others that if they saw you in that state, that they would have questions?*

*R: Aaaaaa they’ll have questions but if it happens it’ll be easy to explain.*

*I: And what is it that is to be more explained?*

*R: What to explain is, many of them may think that mmmmhhh... she is using the pills... is she infected? I’ll tell them no. If I have infections, I’ll tell them the truth. But if I don’t have them, I have the ability to tell them that these medicines aren’t like that... they help in prevention of infection...”*

*(25-year-old, baseline, Tanzania)*

Another participant noted:

*“Previously it was hard for me to be open about it because people had misconceptions about it thinking maybe one is infected. However, now it is clear to most.”* (*24-year-old, baseline, Tanzania*)

## Discussion

We found that anticipated or experienced PrEP stigma manifested in two ways among our study participants: as HIV-related stigma, when participants were assumed to be living with HIV due to the similarity of the PrEP pill bottles to HIV treatment pill bottles, and as sexual behavior stigma, when participants were assumed to be promiscuous or immoral for engaging in sexual activity outside the confines of acceptable gender norms in their contexts. In Tanzania, the sexual behavior stigma appeared more linked with the participant’s engagement in transactional sex during their work at bars or cafes, whereas in South Africa, it was more linked with perceptions that young women’s behavior did not conform with sexual behavior norms for young women. Resilience to stigma was observed in both countries, with participants reporting strategies to avoid disclosure of PrEP use to family and partners, such as hiding pills or taking them when other family members or partners were not around. In both countries, many participants reported taking PrEP despite stigma, as the desire to avoid HIV infection superseded concerns of experiencing stigma.

Like previous studies conducted among AGYW in South Africa, we found that PrEP pills and medication bottles were often mistaken for HIV treatment, leading to parent or partner assumptions that the AGYW was living with HIV [17–19]. This is consistent with quantitative data from our study that found that individuals who reported fear and shame about living with HIV were more likely to report anticipated PrEP stigma [20]. While using different containers for PrEP dispensation or introducing discreet long-acting PrEP methods could mask their association with ART medications, this does not address the underlying issue that many people in the community still hold stigmatizing attitudes towards people living with HIV. Instead, implementing evidence-based interventions at the community level to reduce HIV stigma should be considered [21].

Our findings around the common linkage of AGYW PrEP use with perceptions of promiscuity also aligns with previous research in South Africa and Kenya [17,19,22]. Indeed, this type of sexual behavior stigma, appears to be the most pernicious, as it interferes with the ability of AGYW to access sexual and reproductive health services, including PrEP [15]. While many of our study participants were able to persevere and come up with strategies to enable them to take PrEP, such resilience may not be possible for younger adolescents or when AGYW are accessing PrEP outside of a study setting.

Conducting community-wide education as PrEP is introduced and scaled could be beneficial in reducing sexual behavior stigma, normalizing and increasing support for PrEP as a health promotion strategy, which may in turn facilitate uptake and continuation of PrEP among AGYW [23,24]. In our findings, PrEP sexual behavior stigma originated at the community level and influenced PrEP use through interpersonal relationships and provider-patient interactions. It was clear through the transcripts in both South Africa and Tanzania that community members did not know what PrEP was and were not talking about it openly. This placed the responsibility for educating family members, partners, and peers on the AGYW themselves. While our empowerment intervention showed no effect on PrEP continuation at 6 months, participants experienced benefits from participation, including having a safe space to problem-solve around experienced PrEP stigma [25]. For some participants, they were resilient when faced with stigma, they were empowered to take ownership over their health, and this did not deter them from initiating use. However, most participants from our study were 18 years of age or older and younger adolescent girls may face more difficulty.

Similar to Nyblade et al., we found that strategies to address clinic-based stigma towards AGYW are also needed [15]. Not only did the study participants mention that in the past they experienced judgement from HCP when accessing sexual and reproductive health care, there is also evidence in the literature that age bias exists among providers when it comes to these services. In a study in Nigeria, researchers found that minimum age bias was the most common form of bias among providers, with 15 years of age being the youngest to whom they would offer contraceptives [26]. Furthermore, studies have shown that clinics offering sexual and reproductive health services that were more youth-friendly had increased service use and improved clinic experience for adolescent clients [27,28]. The study in Zambia pointed out that community acceptance of adolescents accessing sexual and reproductive health services is influential to adolescent health-seeking behaviors [28]. This affirms the need for community-wide education in conjunction with education for health facility staff to maximize the effectiveness of these education-based efforts. At the clinic level, providers may benefit from sensitization training on provision of non-judgmental adolescent sexual and reproductive health services, as well as PrEP use, as recommended by WHO [29].

Our study drew strength from the inclusion of interviews with 69 participants, community stakeholders and HCP in both South Africa and Tanzania. A few limitations must be acknowledged, however. These interviews were conducted prior to national introduction of PrEP in programs and may not be generalizable to settings where oral PrEP has become normalized. Several discreet long-acting PrEP methods are now available which may reduce the HIV-related stigma associated with PrEP. Nevertheless, gendered norms regarding expectations of women’s sexual behavior in many settings are still deeply entrenched, and PrEP stigma is likely to persist making these findings relevant. Additionally, we did not include parents or partners in our study, so we are not able to compare responses provided by AGYW about the attitudes and behaviors of their parents and partners surrounding PrEP use. Future research should consider examining the interpersonal connections among AGYW and their parents and partners to inform the development of interventions to minimize stigma and enhance support for PrEP use.

Overall, our findings suggest that anticipated stigma was a common yet surmountable concern of EMPOWER study participants. Negative attitudes about HIV and the sexual behavior of young women were common, which may hamper efforts to scale-up this important prevention tool. Future PrEP implementation should be paired with community-wide education to raise awareness and position PrEP as a responsible, lifestyle choice for young people seeking to protect their health [30]. Training for health care providers is vital to ensure that youth-friendly, gender-sensitive and stigma-free sexual and reproductive health services are available for AGYW to promote PrEP and other HIV prevention methods.
